# Introduction of nicotine analogue-containing oral pouch products in the United States

**DOI:** 10.18332/tpc/195621

**Published:** 2024-11-25

**Authors:** Sven E. Jordt, Sairam V. Jabba

**Affiliations:** 1 Department of Anesthesiology, Duke University School of Medicine, Durham, United States; 2 Cancer Prevention and Control, Duke Cancer Institute, Duke University School of Medicine, Durham, United States; 3 Yale Tobacco Center of Regulatory Science, Department of Psychiatry, Yale School of Medicine, New Haven, United States

**Keywords:** addiction, non-cigarette tobacco products, tobacco industry, nicotine, nicotine analogue

## Abstract

In 2023, 6-methyl nicotine (6MN), a synthetic nicotine analog, was introduced in US-marketed electronic cigarette products advertised as exempt from regulation. It is unknown whether the use of 6MN has spread to other product categories. Industry reports, patent and trademark databases were searched for 6-methyl nicotine products. Identified trademarks ‘Metatine’, ‘Nixotine’, ‘Imotine’ were used to search for US-marketed products. Ingredient information was compared to US market-leading products, and safety warnings and regulatory statements were assessed in context with US state and federal regulations. Two US-based oral pouch brands, ‘MG’ and ‘Hippotine’ pouches, were identified in August 2024, advertised to contain ‘Imotine’-trademarked 6MN. MG Pouches are marketed in four flavors, and ‘Hippotine’-branded pouches are marketed in two flavors, likely representing banned flavor characterization in US state jurisdictions such as California. 6MN contents range 8–25 mg. Otherwise, the ingredient lists were almost identical across both product lines. Products list extensive addiction and health warnings, including warnings not to operate vehicles. Vendors state that these are not tobacco products, which implies that federal and state tobacco regulations do not apply. The spread of nicotine analogs to additional product categories, such as oral pouches, is concerning, especially given the high declared 6MN contents exceeding nicotine in popular US-marketed oral nicotine pouch products. Legislators and regulators need to provide certainty about the regulatory status of nicotine analogs to prevent further erosion of tobacco flavor bans and other regulations.

## INTRODUCTION

In 2023, 6-methyl nicotine (6MN), a synthetic nicotine analog, was introduced in US-marketed electronic cigarette products advertised as exempt from FDA regulation^1^. Since then, several disposable e-cigarette products and refill liquids containing 6MN appeared on the US market, with the compound branded ‘Metatine’ or ‘Nixotine’, the latter mixed with nicotinamide^2^. An additional trademark, ‘Imotine’, was registered for 6MN, marketed by the company Novel Compounds^3-5^. In preclinical studies, 6MN was found to be at least three times more potent than nicotine at eliciting characteristic behaviors, with a lower median lethal dose, raising concerns about increased addictiveness and toxicity^1^. Its effects on humans remain to be assessed.

It is unknown whether the use of 6MN has spread to other product categories, including oral nicotine pouches (ONP) or other oral products such as gums and lozenges. ONP represents a new tobacco product category with rapidly growing sales in the US and worldwide^6^. Here, we aimed to identify US-marketed nicotine analogue-containing products in categories beyond electronic cigarettes.

Industry reports, Google Patents, and the United States Patent and Trademark Office (USPTO) database were searched for information, patent applications, and trademarks for 6-methyl nicotine products. Identified trademarks ‘Metatine’, ‘Nixotine’, ‘Imotine’ and ‘6-methyl nicotine’ (and variations thereof) were used as search terms to identify US-marketed 6MN products. Identified ONP products were purchased from web merchants to confirm availability. Information on ingredient content was reviewed and compared with ingredients in market-leading ONP products. Marketing and safety claims were reviewed, and regulatory statements were assessed in context with regulations of US states, especially California, and federal laws and regulations.

## COMMENTARY

We used the search terms ‘Metatine’, ‘Nixotine’ or ‘Imotine’ to search for newly introduced 6MN-containing products marketed by web merchants in the US. In August 2024, we identified two US-based brands, ‘MG’ and ‘Hippotine’ pouches, advertised to contain ‘Imotine’. No ‘Metatine’, or ‘Nixotine’ pouch products were identified, and no products of other categories (gums, lozenges, etc.) with nicotine analogs were identified.

‘MG’ pouches are marketed by Upperdeckys.com, a vendor of caffeine-containing ‘Energy’ pouches. ‘Hippotine’ pouches are marketed by the web merchant Happyhippo.com, a vendor of Kratom^7,8^. Kratom is an herbal extract designated by FDA as an unapproved product with strong consumer warnings of the risk of serious adverse events^9^.

MG Pouches are marketed in four flavors: Cool Mint (8 mg Imotine per pouch), Buzzin Berry (8 mg), Wintergreen (15 mg), and Orange Creamsicle (25 mg) (Table 1). Hippotine-branded pouches are marketed in two flavors: Guava Juice (15 mg Imotine per pouch) and Wintergreen (25 mg) (Table 1). Fruit and sweet dessert flavors (Buzzin Berry, Orange Creamsicle, Guava Juice) are especially known to appeal to youth and young adult nicotine pouch users^10^.

**Table 1:** Nicotine contents in pouches of 2024 US market-leading brands (top) compared to newly identified 6MN pouch brands (bottom), with 6MN trademarks, flavors and declared 6MN contents

*Brand*	*Manufacturer*	*Flavor/varieties*	*Nicotine/pouch*
Zyn	PMI/Swedish Match	Cool Mint, Peppermint, Wintergreen, Spearmint, Cinnamon, Coffee, Citrus, Menthol, Smooth, Chill	3 mg 6 mg
Velo	BAT/R.J. Reynolds	Dragon Fruit, Black Cherry, Citrus Burst, Peppermint, Spearmint, Wintergreen, Coffee, Cinnamon	2 mg 4 mg 7 mg
On!	Altria	Mint, Wintergreen, Coffee, Berry, Cinnamon, Original, Citrus	1.5 mg 2 mg 3.5 mg 4 mg 8 mg
**Brand**	**6MN Trademark**	**Flavor**	**6MN/pouch**
MG	Imotine	Cool Mint	8 mg
		Buzzin Berry	8 mg
		Wintergreen	15 mg
		Orange Creamsicle	25 mg
Hippotine	Imotine	Guava Juice	15 mg
		Wintergreen	25 mg

The listed 6-methyl nicotine contents of the newly introduced products are either identical to (8 mg) or by far exceed (15 mg, 25 mg), the maximal nicotine contents of the most popular US-marketed nicotine pouch products (Zyn, Velo, On!), marketed in strengths of 1.5–8 mg (Table 1)^11-14^. The ingredients listed on the back of the cans are almost identical across both product lines, containing: coconut fiber (MG) or coconut coir (Hippotine), vegetable glycerin, palm oil, xylitol, natural flavor, water, Imotine^TM^, sodium carbonate, xanthan gum, stevia, and salt, suggesting they are produced by the same manufacturer.

**Figure 1:**
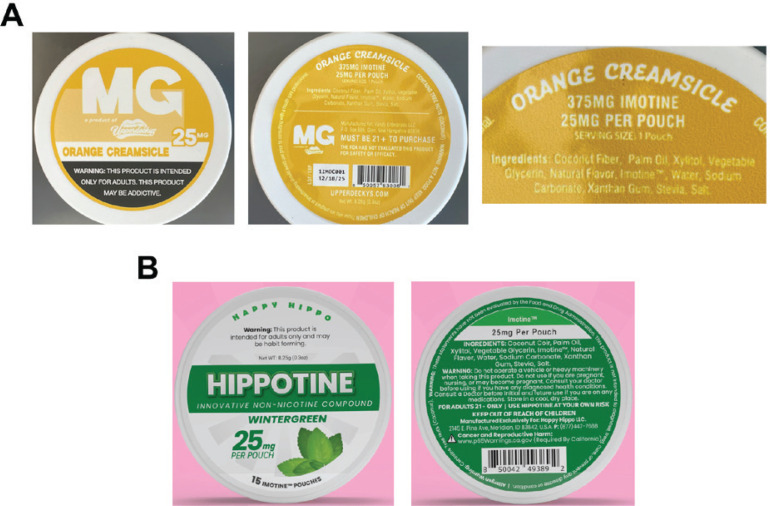
Oral pouch products containing ‘Imotine’-branded 6-methyl nicotine: A) Photographs of Orange Creamsicle-flavored ‘MG’ pouches can, purchased by the authors, labelled to contain 25 mg ‘Imotine’ per pouch. Left: front of can, Middle: back of can, Right: ‘Imotine’ content, ingredients list; B) Wintergreen-flavored ‘Hippotine’ pouches can, labelled to contain 25 mg ‘Imotine’. Left: front of can, Right: back of can.

Vendor websites and product packaging provide extensive addiction and health warnings (Figure 1, Table 2). Some statements refer to the differences in chemistry between 6MN and nicotine and its potential addictiveness and toxicity: ‘Imotine^TM^ is chemically distinct from nicotine. It may still be addictive, may have a toxicity profile similar to Nicotine, and should only be used by current adult tobacco users and never by minors (Persons under the age of 21)’^8^.

**Table 2:** Health warnings and regulatory claims for MG pouches and Hippotine pouches

*Brand*	*Statement*	*Source*
MG	Warning: this product is intended only for adults.	Front label
	This product may be addictive.	Front label
	Contains tree nuts (coconut)	Back label
	Warning: Not a food.	Back label
	Keep out of reach of children	Back label
	Those who are pregnant or breastfeeding should avoid use prior to consulting with a health professional	Back label
	This product is not intended to diagnose, treat, cure, or prevent any disease or condition	Website
	ImotineTM is not considered a tobacco product.	Website
Hippotine	Warning: This product is intended for adults only and may be habit forming	Front label
	Do not operate a vehicle or heavy machinery when taking this product.	Back label
	Do not use if you are pregnant, nursing, or may become pregnant.	Back label
	Consult your doctor before using if you have any diagnosed health conditions.	Back label
	Consult a doctor before initial and future use if you are on any medications.	Back label
	Use Hippotine at your own risk.	Back label
	This product is not intended to diagnose, treat, cure, or prevent any disease or condition	Back label
	Hippotine (Imotine™) is an experimental product currently being studied for its interaction with nicotinic acetylcholine receptors (nAChRs).	Website
	Tobacco-Free, Nicotine-Free, Sugar-Free	Website
	Not listed as a carcinogen by IARC, NTP, NIOSH, or FDA.	Website
	Available to Californians (not subject to flavored pouch restriction).	Website

Health warnings refer to potential risks during pregnancy and breastfeeding, existing health conditions, and possible medication interactions (Figure 1, Table 2).

Additionally, a warning usually not associated with tobacco products is included: ‘Warning: Do not operate a vehicle or heavy machinery when taking this product’ (Figure 1).

Both brands make statements likely aimed to pre-empt regulatory measures. For example: ‘This product is not intended to diagnose, treat, cure, or prevent any disease or condition’, likely addressing potential regulation of the products by FDA as drugs (Figure 1)^8^. The vendor also states that ‘Imotine^TM^ is not considered a tobacco product’, suggesting that tobacco regulatory restrictions do not apply^15^. Hippotine pouches are advertised as: ‘Available to Californians (not subject to flavored pouch restriction)’, aiming to undermine California’s ban on characterizing flavors that extend to oral nicotine pouches^7^. The products are likely illegal in California since new legislation was enacted in the state on 28 September 2024, that deems 6MN a form of nicotine under state tobacco regulatory authority^16^.

### Strength and limitations

The strength of the present study is its use of a wide range of identified trademark names for nicotine analogs in the search for novel products. However, the marketplace is dynamic; new brands and trademarks may have emerged, and some products might have been missed. Conclusions regarding the potential toxicity of the identified products are limited by the incomplete knowledge of 6MN’s health effects in humans and the lack of independent verification of 6MN product contents. Future chemical analysis studies will enable approaches for more accurate human risk assessment.

## CONCLUSION

The spread of nicotine analogs to additional product categories, such as oral pouches, is concerning, especially because of the high listed 6MN contents of the newly introduced products. Given the higher potency of 6MN compared to nicotine in pharmacological studies, regulators need to rapidly assess the potential public health threats associated with these products^1^. Legislators and regulators also need to provide certainty about the regulatory status of nicotine analogs to prevent further erosion of flavor bans and other regulations.

## Data Availability

The data supporting this research are available from the authors on reasonable request.
